# Does Level of Received Intervention Dose Have an Impact on the Effectiveness of the Social Norms Alcohol Prevention Program *The GOOD Life?*

**DOI:** 10.3389/fpubh.2019.00245

**Published:** 2019-08-28

**Authors:** Lotte Vallentin-Holbech, Birthe Marie Rasmussen, Christiane Stock

**Affiliations:** ^1^Unit for Health Promotion Research, Department of Public Health, University of Southern Denmark, Esbjerg, Denmark; ^2^Institute for Health and Nursing Science, Charité Universitätsmedizin Berlin, Berlin, Germany

**Keywords:** adolescents, school-based intervention, implementation, received dose, binge drinking, alcohol-related harms, norm perceptions, cluster-randomized controlled trial

## Abstract

**Background:** Few studies have assessed how social norms messages are perceived and understood by adolescents in secondary school. We examined whether the self-reported level of exposure, satisfaction and recall of a social norms intervention had an impact on the preventive effect of the intervention *The GOOD Life*. Furthermore, we explored which factors were associated with high recall of the intervention.

**Methods:** Data from pupils aged 13–17 years enrolled in a cluster-randomized controlled trial with 18 intervention schools (*n* = 641) and 20 control schools (*n* = 714) were analyzed using multilevel regressions. The intervention provided social norms messages through three different communication elements: classroom feedback session, posters, and web-application. At 3-months follow-up, pupils from the intervention schools were asked about their participation in, their satisfaction with and recall of the intervention. The effects were examined on: overestimation of peer drinking, binge drinking (5 or more drinks on one occasion) and alcohol-related harms.

**Results:** Regards the outcome overestimation of peer drinking higher preventive effect sizes were observed for higher levels of exposure, satisfaction, and recall. Regards the outcome alcohol-related harms preventive intervention effects were observed for medium exposure and higher satisfaction. For binge drinking we found no significant effects for any level of exposure, satisfaction, or recall. Higher levels of satisfaction and exposure, and female sex were associated with better recall of the intervention.

**Conclusion:** For higher levels of self-reported exposure, satisfaction, and retention regarding the social norms messages we observed stronger intervention effects regards several outcomes suggesting that these implementation parameters are important for intervention effectiveness.

**Trial registration:** The trial is registered at Current Controlled Trials with study ID ISRCTN27491960.

## Introduction

Several programmes have been conducted to prevent alcohol misuse and related harms among adolescents (1, 2). Such programmes are often implemented in school-settings because this is regarded as an efficient way to reach a substantial number of young people at risk of initiating harmful drinking behaviors (3–5). While some studies found that school-based programmes were effective, other studies failed to find significant preventive effects among young adolescents (3, 5). Cuijpers (6) suggest that the inconclusive results on effectiveness may be related to the inconsistency of programme content and the diversity of the theoretical frameworks of successful programmes. However, prevention programmes based on the social norms approach (SNA) have shown promising results in relation to reduction of alcohol use in educational settings among both younger (7–10) and older adolescents (11, 12). These SNA interventions place the focus on the healthy behavior of the majority of the target population, hence fosters social cohesion and inclusion rather than pinpointing toward adverse, or anti-social behavior. The key content of the SNA is to illustrate the discrepancy between actual peer behavior and the perceived behavior among peers using positively phrased messages about the level of consumption of the majority of peers (13). The main assumption of the SNA is that by correcting these discrepancies with regard to drinking norms the social pressure on individuals will decrease and this consequently reduces the personal use of alcohol (14). In Denmark, a recently conducted cluster-randomized controlled trial testing the school-based social norms intervention *The GOOD Life* (in Danish: Det GODE Liv) showed a significant reduction of Danish pupils aged 13–17 years that overestimated their peers' lifetime binge drinking and a decrease in the number of reported alcohol-related harms, while no significant effect on binge drinking was found (10).

Previous research suggests that the effectiveness of preventive interventions in schools may be affected by implementation parameters such as acceptance of core components, duration and reach of the intervention, and by mode of delivery (6, 15–17). For example, Bewick et al. (11) found an additional reduction in alcohol use among university students who reported a higher level of exposure to the social norms intervention “Unitcheck.” Some studies found that preventive effects were related to the retention of the information given during the intervention period (18). Further, the Elaboration Likelihood Model (ELM) (19) suggests that appealing messages can be categorized as peripheral cues, which have the potential to affect the acceptance of a message and in turn persuade the receiver of the argument delivered. Hence, it can be anticipated that high appeal of the intervention and messages would increase pupils' attention to the programme content and thereby facilitate their ability to better retain the core messages (15, 20).

To support pupils in processing and comprehending the intervention content studies have suggested that school-based prevention programmes should actively engage pupils through interactive delivery methods such as “hands-on” workshops and web-based quizzes (6, 16, 21). Therefore, we assumed that the effectiveness of a social-norm based intervention requires that pupils fully understand the social norms messages delivered and can identify them after the completion of the programme. The relevance of pupils' recall of the social norms messages on the intervention effects has received only limited attention (1, 18) and Dempsey, McAlaney and Bewick (22) warrant more research regards user experiences engaging with SNA feedback. Only a few studies have assessed in detail how social norms messages are perceived and understood by adolescents in secondary schools and if a higher self-reported exposure level of the intervention could provide stronger intervention effects (3, 6).

The current study aimed to investigate whether the self-reported level of exposure to, satisfaction with and recall of the social norms messages in *The GOOD Life* intervention were moderators of the intervention effects measured by the outcomes: pupils' perception of peer lifetime binge drinking, frequency of binge drinking and alcohol-related harms. Further, it was explored if age and sex, as well as the self-reported level of exposure to and satisfaction with the intervention were associated with the recall of the social norms messages.

## *The GOOD Life* Intervention

The intervention *The GOOD Life* was developed by the project team based on research experience from a previous SNA study (23) and on recommendations how to apply the SNA in interventions (13). All communication elements were pre-tested in one school class and feedback from pupils was used to optimize the intervention design and content.

The content of the intervention provided normative feedback by tailoring social norms messages for each grade at the participating schools based on the results from all responses to questions about alcohol consumption and approval of use from the baseline survey (*n* = 2325). During the intervention period of 8 weeks, the social norms messages were delivered to pupils through three different communication elements: face-to-face (classroom feedback session), printed (posters), and interactive media (web application).

**Figure 1:**
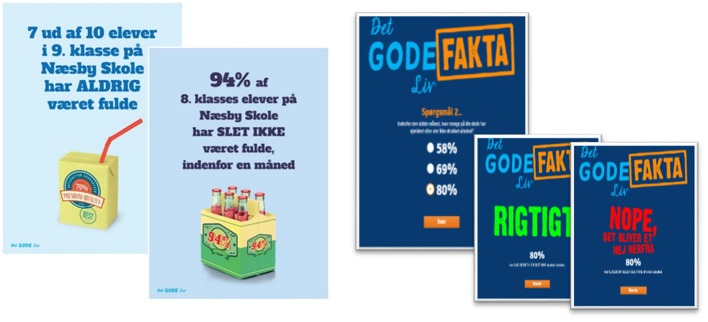
Posters and web application displaying social norms messages tailored for each school and grade used in the intervention *The GOOD Life*.

The tailored messages were phrased with focus on the healthy behavior of the majority in order to deliver factual information of peer behavior and attitudes toward alcohol use in a positive manner, e.g.,“8 out of 10 pupils in 8th grade at [school name] have NEVER been drunk” (Figure 1). All messages were delivered by members of the research team who were not blinded to the allocation of the schools (24).

The feedback session was a single classroom activity of 45–60 min duration where pupils were asked to use a Student Response System (SRS) that engaged them in a poll regarding four to five questions about what they think how much their peers consume alcohol or approve its use. The session was led by a trained member of the research team. Using the SRS with their smartphones each pupil in the classroom answered the questions by choosing from three potential answers. Thus, the SRS displayed what the pupils collectively assumed to be the true drinking norm or level of peer approval of drinking in their peer group. This norm was compared with the results from the baseline survey that measured the actual prevalence of drinking or approval of drinking in their grade and school. Discrepancies between perceived and actual norms were discussed with pupils.

After the feedback session, each school received four to six posters with additional social norms messages. The graphic design relied on text and pictures displaying the messages, but without stigmatizing any individuals and using a distinctive color scheme to create a “look” similar to popular adverts (see Figure 1). Teachers were asked to display the posters in areas where pupils would see them every day for the remaining intervention period. Additionally, pupils were encouraged by their teachers to access a web application as depicted in Figure 1, which was a website to access on their computers or smartphones containing a multiple-choice quiz where pupils should choose the correct answer out of three answering options to questions about how many of their peers use or approve alcohol and they received an answer about the correct response option (24). The correct answers were based on data from the baseline survey.

## Materials and Methods

### Study Sample

Between February 2015 and August 2016, 46 out of 135 secondary public schools in the Region of Southern Denmark were enrolled in the study. All school principals were invited by mail and 46 (34%) agreed to the study. Reasons for declining the invitation were busy school schedules and having participated in other drug prevention programmes. Pupils aged 13–17 years (grade 8 and 9) were invited to participate in the study by their teachers and active parental consents were obtained.

Eight schools were excluded from the study because they did not fulfill the trial protocol (e.g., missing deadlines and/or consent), allowing 2,325 pupils from 38 schools to be included in the baseline survey. Follow up attrition was low with 228 pupils (10%) not completing the follow-up survey because their school class dropped out and 120 pupils (5%) not completing because they were absent. Unfortunately, 622 pupils (27%) were excluded from the analyses because the generated identification code for each individual respondent could not be replicated in the follow-up survey. Higher attrition rates were found for boys [${\text{Chi}}_{\left(1\right)}^{2}$ = 4.1, *p* = 0.044] and older pupils [${\text{Chi}}_{\left(4\right)}^{2}$ = 25.3, *p* < 0.001]. Also, higher attrition rates were found for the baseline measures of: lifetime binge drinking [${\text{Chi}}_{\left(1\right)}^{2}$ = 26.5, *p* < 0.001), binge drinking [${\text{Chi}}_{\left(10\right)}^{2}$ = 42.3, *p* < 0.001] and alcohol-related harms [${\text{Chi}}_{\left(14\right)}^{2}$ = 43.6, *p* < 0.001] (10). In total 970 pupils (42%) were lost to follow-up leaving 1,355 pupils from 38 schools to be included in the final analyses where 641 pupils were allocated in the intervention schools and 714 pupils in the control schools. We compared pupils in the intervention schools with pupils in the control with regard to sex, age, grade, and perceived family affluence at baseline. Pupils in the intervention schools were significantly older than pupils in the control schools, but did not differ in the other characteristics (10). Therefore, all analyses were adjusted for age.

Due to an error in the 2016 data collection data for alcohol-related harms were only collected for 540 pupils. Attrition analysis was conducted for the group of pupils that did not have the option to respond to these questions (*n* = 560). Pupils not included in the analyses with alcohol-related harms as outcome were mainly allocated in the control group [${\text{Chi}}_{\left(1\right)}^{2}$ = 57.99, *p* < 0.001]. Baseline differences between responders and non-responders were only found for age [${\text{Chi}}_{\left(4\right)}^{2}$ = 32.51, *p* < 0.001] and overestimation of peer lifetime binge drinking [${\text{Chi}}_{\left(1\right)}^{2}$ = 6.86, *p* = 0.009]. Data imputation was not performed, because it is not a recommended procedure for dependent variables and for data not missing at random (25).

### Study Design

The study was designed as a cluster-randomized controlled trial where schools were randomly allocated to either the intervention group or the control group (24). Using the Microsoft Excel randomization function, the allocation was carried out by a researcher not blinded to the identity of the schools, but with limited knowledge about the schools.

Using online questionnaires, data were collected on pupils' alcohol use and perception of peer alcohol use 1–2 weeks before the intervention start (baseline) and 3 months after the first survey (follow-up). In both surveys pupils were informed that data were collected anonymously and treated confidentially. All measures were self-reported by pupils. The data collection was facilitated by the corresponding teachers in a classroom setting where the questionnaire was accessed by self-registration at the survey website. The intervention was delivered to schools in the intervention group 1 or 2 weeks after the baseline survey and to the schools in the control group after the 3-months follow-up survey was completed. In the 3-months follow-up survey pupils in the intervention group (*n* = 641) were asked about the implementation parameters (exposure level, satisfaction and recall) related to the intervention.

An overview of the entire study, including a description of the school setting, the recruitment of schools for the study and details on the intervention, is provided in the study protocol (24, 26). The trial was approved by the Ethical Committee of the Region of Southern Denmark (Project-ID: S-20140185) and is registered at Current Controlled Trials with study ID: ISRCTN 27491960.

### Measures

Alcohol use and perceived alcohol drinking among peers were measured in both the baseline and follow-up questionnaire. The baseline questionnaire covered demographic information on age, grade and sex and was pre-tested among pupils aged 14–15 years. To assess the implementation parameters regarding pupils' level of exposure to the intervention, their satisfaction with and their recall of the intervention, corresponding questions were included in the follow-up questionnaire for the pupils in the intervention group (*n* = 641).

#### Measurement of Outcome Variables

Outcome variables were measured for all 1,355 pupils in both the baseline and follow-up questionnaire using the following measures.

#### Overestimation of Peer Lifetime Binge Drinking

To examine changes in perceived social norms the actual prevalence of lifetime binge drinking for each grade and school was calculated and used as the measure of the actual norm regards binge drinking. Then pupils were asked to rate the percentages (0–100%) of peers of their own school who had ever had 5 or more drinks on one occasion (binge drinking) (23, 27). Similar to methods used in other studies (28) we constructed a measure from the two variables mentioned above and classified pupils who estimated the prevalence of binge drinking among peers of their own grade to be more than 10% above the actual prevalence as having overestimated the prevalence of lifetime binge drinking among their peers. This resulted in a dichotomous variable for overestimation: no (coded 0) and yes (coded 1).

#### Frequency of Binge Drinking Within the Last 30 Days

Pupils were asked to identify the number of occasions they had been drinking 5 or more drinks on one occasion (binge drinking) in the last 30 days (23, 27, 29). The response options were never to more than 10 times.

#### Alcohol-Related Harms

A summary measure based on a scale used in the SNIPE study (23) consisting of 10 items on different consequences related to alcohol use was used to estimate the number of alcohol-related harms. To tailor the scale to this study population, we included five items from the Danish youth survey MULD (27). Each item had response options indicating whether pupils did or did not experience the specific consequence. The items covered questions such as “have you ever experienced discomfort or had a hangover?,” “have you ever got into a fight?, and have you ever got in trouble with the police?” All 15 items were combined into an additive score ranging from 0 to 15 (Cronbach's alpha: 0.75).

### Measurement of Implementation Parameters (Effect Moderators)

The follow-up survey among pupils in the intervention group (*n* = 641) assessed self-reported exposure to, satisfaction with and recall of all three communication elements as separate parameters for the implementation of the intervention.

### Level of Exposure to *The GOOD Life*

The self-reported exposure level was assessed using two measures. (1) Asking pupils if they remembered having participated in the feedback session, having seen the posters and/or having used the web-application resulted in a measure of exposure to one, two or three communication elements. (2) Pupils that indicated that they could remember having seen posters were asked about the number they could recall having seen (1–10 posters). Based on quartiles, four categories were used for the analyses: 1–2, 3–4, 5–6, and 7–10 posters.

### Level of Satisfaction With *The GOOD Life*

Self-reported satisfaction with the feedback session, posters and web application was assessed using a measure for each element asking pupils to indicate on a 5-point scale “How satisfied are you with the feedback session/posters/web-application?” The scale ranged from very dissatisfied (1) to very satisfied (5). For the analyses, the five response options were divided into three categories. Pupils answering very dissatisfied and dissatisfied were collapsed into the category *Low satisfaction*, pupils answering somewhat satisfied was categorized as *Okay satisfaction* and the two last options were categorized as *High satisfaction*.

### Level of Recall of the Messages in *The GOOD Life*

Self-reported retention of social norms messages from the feedback session, from posters and from the web-application was assessed separately by calculating the number of messages correctly recalled at the 3-months follow-up survey. The measure was constructed to identify if pupils could distinguish between four correct and four incorrect statements regards the intervention content. From a list of eight statements (four social norms messages and four alcohol/risk related statements), pupils were asked to indicate those used in *The GOOD Life* intervention. Pupils received one point when they answered “yes” to the correct social norms messages and one point if they answered “no” to the incorrect alcohol statements, resulting in a total maximum score of eight correct answers for each communication element. For the analyses, the score was divided by median split into *Low recall* (score: 0–5) and *High recall* (score: 6–8) for all three communication elements.

### Data Analyses

For the descriptive statistics depicted in Table 1 means were calculated for age, binge drinking and alcohol-related harms and frequencies for sex, grade and overestimation of peer binge drinking.

**Table 1:** Sample characteristics by control and intervention groups and descriptive statistics of the implementation parameters for the intervention group at 3-months follow-up.

	**Control group *n* = 714**		**Intervention group *n* = 641**	
	***n***	**%**	***n***	**%**
Boys	322	45.1	301	47.0
Girls	392	54.9	340	53.0
Grade 8	348	48.7	338	52.7
Grade 9	366	51.3	303	47.3
Age, mean (SD)	14.7	(±0.6)	14.8	(±0.7)
**Outcome variables**				
Overestimation of peer lifetime binge drinking	323	45.8	241	38.1
Binge drinking in the last 30 days, mean (SD)	1.1	(±2.4)	1.0	(±2.3)
Alcohol-related harms^a^, mean (SD)	1.6	(±2.2)	1.4	(±2.2)
**Participation in *The GOOD Life* elements**				
Feedback session			523	81.6
Posters			343	53.5
Web-application			211	32.9
**Level of self-reported exposure to *The GOOD Life***				
Elements				
One element			159	29.6
Two elements			220	41.0
Three elements			158	29.4
Posters				
1–2 posters			67	19.9
3–4 posters			101	30.0
5–6 posters			107	31.8
7–10 posters			62	18.4
**Level of satisfaction with *The GOOD Life***				
Feedback session				
Low satisfaction			35	6.8
Okay satisfaction			223	43.6
High satisfaction			253	49.5
Posters				
Low satisfaction			26	7.7
Okay satisfaction			159	47.2
High satisfaction			152	45.1
Web-application				
Low satisfaction			12	5.8
Okay satisfaction			105	50.7
High satisfaction			90	43.5
**Level of recall of *The GOOD Life***				
Feedback session				
Low recall			295	62.9
High recall			174	37.1
Posters				
Low recall			166	51.9
High recall			154	48.1
Web-application				
Low recall			121	64.4
High recall			67	35.6

a *Only measured among 540 pupils with lifetime alcohol use due to an error in the electronic questionnaire in the 2016 data collection*.

Due to the hierarchical study design data were regarded as dependent because pupils were clustered within schools. Hence, multilevel logistic regression models for the binary outcome overestimation of peer drinking and negative binominal regression analyses for the continuous outcomes binge drinking and alcohol-related harms were used to investigate the effects of the intervention vs. control group on the three outcome variables. All models were fitted using two levels: school and pupils. Baseline values for the corresponding outcome variables were controlled for by including them into the models as well as the co-variables age and sex. Cluster-size in the final analyzed sample varied from 2 to 91 pupils but did not significantly differ between control and intervention groups [Pearson ${\text{Chi}}_{\left(8\right)}^{2}$ = 9.874, *p* = 0.274].

To investigate, if implementation parameters moderate the intervention effect, a set of multi-level models was run with increasing levels of the implementation parameters for exposure, satisfaction and recall as independent dummy variables using the control group as reference. In order to assess the impact of increasing exposure to the intervention on effectiveness of *The GOOD Life* the analyses were conducted for pupils having received 1, 2, or 3 communication elements compared to 0 as reference. The analyses were not conducted for each communication element (feedback session, poster, web application), because the sum of the elements is regarded as increasing exposure and not the quality of the single elements and because the majority of the sample (70%) received communication elements in combination.

To answer the second objective of this study, factors independently associated with the dependent variable high recall of the social norms messages were analyzed with a multilevel logistic regression model including sex, grade, exposure level, and satisfaction with school as random effect. We used recall of messages from the feedback session for this analysis, because the number of pupils participating in the feedback session was highest and provided the best statistical power for the analysis of associated factors.

All analyses were performed using the statistical package Stata/IC 15.0.

## Results

The analyzed study population comprised 1,355 pupils with a mean age of 14.8 (SD ± 0.67) years. The percentage of girls was 54 and 49% of the pupils were in grade 9 (Table 1). The difference between intervention and control group was significant for overestimation of peer lifetime binge drinking and for alcohol-related harms, but not for frequency of binge drinking (Table 2).

**Table 2:** Impact of implementation parameters on overestimation of peer drinking, frequency of binge drinking and alcohol-related harms as outcomes of *The GOOD Life*.

	**Overestimation of peer lifetime binge drinking (*n* = 1355)**		**Binge drinking in the last 30 days (*n* = 1355)**		**Alcohol-related harms (*n* = 540)**	
	**OR^a^**	**95%CI**	**Coef.^a^**	**95%CI**	**Coef.^a^**	**95%CI**
**Main intervention effects**						
Control group	Ref.		Ref.		Ref.	
Intervention group	**0.52**	**0.33; 0.83**	0.004	−0.16; 0.17	–**0.27**	–**0.53; –0.02**
**Level of exposure to *The GOOD Life* elements**						
Control group	Ref.		Ref.		Ref.	
One element	0.64	0.36; 1.13	0.01	−0.19; 0.22	−0.10	−0.43; 0.23
Two elements	**0.41**	**0.24; 0.69**	−0.07	−0.27; 0.13	**−0.47**	**−0.79; −0.15**
Three elements	**0.45**	**0.25; 0.79**	−0.003	−0.21; 0.21	−0.14	−0.50; 0.22
**Level of exposure to posters**						
Control group	Ref.		Ref.		Ref.	
1–2 posters	**0.26**	**0.12; 0.56**	−0.04	−0.30; 0.21	0.07	−0.43; 0.57
3–4 posters	0.55	0.29; 1.03	−0.06	−0.30; 0.17	−0.02	−0.42; 0.37
5–6 posters	0.59	0.32; 1.11	0.09	−0.14; 0.31	−0.28	−0.68; 0.12
7–10 posters	0.50	0.23; 1.06	−0.21	−0.51; 0.08	0.11	−0.40; 0.62
**Level of satisfaction with *The GOOD Life***						
Control group	Ref.		Ref.		Ref.	
Low (feedback session)	0.51	0.21; 1.22	0.09	−0.22; 0.42	−0.24	−0.83; 0.34
Okay (feedback session)	**0.45**	**0.27; 0.75**	−0.13	−0.33; 0.06	**−0.37**	**−0.70; −0.05**
High (feedback session)	**0.50**	**0.30; 0.82**	0.04	−0.15; 0.23	**−0.33**	**−0.64; −0.01**
Control group	Ref.		Ref.		Ref.	
Low (posters)	0.59	0.22; 1.58	0.22	−0.12; 0.56	0.01	−0.70; 0.73
Okay (posters)	**0.53**	**0.30; 0.93**	−0.01	−0.22; 0.19	−0.35	−0.73; 0.02
High (posters)	**0.43**	**0.24; 0.77**	−0.16	−0.38; 0.05	−0.01	−0.39; 0.37
Control group	Ref.		Ref.		Ref.	
Low (web-application)	0.56	0.14; 2.25	0.10	−0.42; 0.63	−0.86	−1.92; 0.20
Okay (web-application)	**0.39**	**0.20; 0.76**	−0.05	−0.28; 0.18	**−0.56**	**−1.02; −0.10**
High (web–application)	**0.36**	**0.18; 0.74**	−0.02	−0.26; 0.22	0.04	−0.39; 0.48
**Level of recall of *The GOOD Life***						
Control group	Ref.		Ref.		Ref.	
Low (feedback session)	**0.52**	**0.32; 0.84**	−0.03	−0.21; 0.15	−0.16	−0.41; 0.08
High (feedback session)	**0.31**	**0.18; 0.54**	−0.04	−0.24; 0.17	−0.31	−0.64; 0.02
Control group	Ref.		Ref.		Ref.	
Low (posters)	0.58	0.33; 1.02	−0.02	−0.23; 0.18	0.01	−0.32; 0.34
High (posters)	**0.36**	**0.19; 0.66**	−0.09	−0.31; 0.14	−0.14	−0.50; 0.22
Control group	Ref.		Ref.		Ref.	
Low (web–application)	**0.41**	**0.22; 0.77**	−0.04	−0.27; 0.20	−0.17	−0.55; 0.20
High (web–application)	**0.39**	**0.18; 0.85**	0.01	−0.27; 0.29	−0.04	−0.48; 0.41

a *Estimates with corresponding 95% confidence interval (CI) based on multilevel logistic or negative binominal regression models with the control group as reference. All models were adjusted for baseline values of the corresponding outcomes, age, and sex. Schools were included as random effect. Bold typeface indicates significant values (p < 0.05)*.

Table 1 shows that the vast majority (82%) of pupils in the intervention group participated in the feedback session, 54% reported having seen the posters, whereas only 33% used the web-application. In total 84% (*n* = 537) of the pupils in the intervention group reported having seen or participated in at least one of the intervention elements. Among these, 41% reported having participated in two communication elements. In general, the pupils were satisfied with both the feedback session, posters, and web-application. The percentage of pupils with high recall of intervention messages was higher for posters (48%) than for the feedback session (37%) and for the web-application (36%).

Table 2 shows the impact of the implementation parameters on the three outcomes. Exposure to a higher number of intervention elements resulted in lower odds ratios regarding overestimation, while among pupils exposed to only one element the intervention did not lead to a significant reduction in overestimation of peer binge drinking. Regards the outcome alcohol-related harms a medium level of exposure (two elements) showed a significant intervention effect, while we found no significant intervention effects on binge drinking for other levels of exposure. No significant intervention effect was noted for pupils exposed to a higher number of posters for any of the outcomes. The only significant effect was observed regards reduced overestimation among those exposed to only 1–2 posters.

Regarding the level satisfaction with the intervention elements the analyses showed significant intervention effects on overestimation among pupils with okay and high satisfaction with all three communication elements, but not for low level of satisfaction. For the outcome alcohol-related harms a significant intervention effect was found among pupils reporting okay and high satisfaction with the feedback session and ok level of satisfaction with the web application, but not for those with low level of satisfaction.

The analyses showed that better recall (recalling 6–8 messages correctly) of the social norms messages from all three communication elements resulted in significant intervention effects on overestimation of peer lifetime binge, but not on the outcome alcohol-related harms.

Regarding the outcome of binge drinking the implementation parameters did not have an impact on intervention effects for any level of the implementation parameters.

The results of the analysis of factors associated with high recall of the intervention messages are depicted in Table 3. High level of satisfaction was associated with higher recall of messages in the feedback session. Pupils exposed to three intervention elements were more likely to have a better recall of the messages from the feedback session. Grade was not significantly associated with the level of recall, but girls were more likely to recall the intervention messages correctly than boys.

**Table 3:** Factors associated with high recall of the content in the interactive feedback session.

	**High recall of the feedback session (*n* = 174)**	
	**OR^a^**	**95%CI**
Boys	Ref.	
Girls	**1.87**	**1.24–2.82**
Grade 8	Ref.	
Grade 9	1.48	0.97–2.25
One element	Ref.	
Two elements	1.42	0.81–2.48
Three elements	**1.88**	**1.01–3.49**
Low satisfaction	1.01	0.40–2.53
Okay satisfaction	Ref.	
High satisfaction	**1.71**	**1.12–2.60**

a *Odds Ratios with corresponding 95% confidence interval (CI) based on a multilevel logistic regression model with school included as random effect. Bold typeface indicates significant values (p < 0.05)*.

## Discussion

The analyses of data from this cluster-randomized controlled trial assessed the impact of several implementation parameters on the effect of the intervention *The GOOD Life* as effect moderators. The analyses showed overall stronger intervention effects when the level of exposure to, the satisfaction with or the level of recall of the social norms messages was high. This is concurrent with alcohol intervention studies among university students in the United Kingdom (11) and North America (17, 30) that found that alcohol prevention programmes with several components produced stronger effects. Even though the intervention effect remained insignificant for frequency of binge drinking, the results demonstrated that exposure to more than one intervention element (e.g., feedback session plus posters) overall increased the preventive effect of *The GOOD Life* regards the other outcomes and lead to reduced overestimation of binge drinking among peers. The preventive effects did not improve among pupils that recalled having seen a higher number of posters. This finding suggests that displaying very high numbers of posters might be less relevant for the intervention to be effective. However, because an effect was observed when pupils participated in two out of three intervention elements, but not when participating in only one element, the posters certainly can be regarded as a reminder or booster that consequently increase the exposure level and overall intervention dose. Thus, improving compliance of schools and teachers to clearly display at least some posters with social norms messages would have the potential to increase the effect size. The same is true for encouraging pupils to use the web application. Our trial showed that the web application was used by pupils in some schools, but not in others, indicating that the commitment of teachers is important to enhance the use of this social norm communication element and to secure high intervention exposure.

Miller and Prentice (16) suggest in their review that the credibility of the messages is essential for measuring effects of a social norms intervention and that groups sharing a salient social identity may be more responsive to social norms messages. The social norms messages in *The GOOD Life* were designed to be group-specific by distinctly including references to a proximal peer group. The messages were based on self-reported data which according to the SNA would be perceived by pupils as relevant and accepted as credible (13, 14). Previous research (19, 31) has demonstrated that the acceptance of a message was related to the perceived overall appeal of the message. Therefore, appeal may have the potential to affect the perceived credibility of the arguments delivered by the social norms messages. Our study showed that about half of the pupils (49.5%) found the feedback session very appealing and the odds ratios indicated stronger preventive effects among pupils reporting being satisfied with the intervention *The GOOD Life*. Also, the findings showed that pupils' satisfaction with *The GOOD Life* was positively associated with better recall of the social norms messages reflecting that perceived high appeal was related to higher retention of the intervention. We suggest that our analyses provide some support for the hypothesis based on the ELM proposing that appeal acts as a peripheral cue and is associated with increased effectiveness of school-based prevention programmes (19).

Moreover, studies have suggested that high appeal of the intervention components could increase pupils' attention to the messages delivered and in turn help them to comprehend the key arguments better (15, 20). In the current study, stronger intervention effects on overestimation of peer lifetime binge drinking were found among pupils with better recall of the social norms messages. This is consistent with the findings by Jouriles et al. (18) who found a relationship between intervention effects and the retention of information delivered during the intervention period. As hypothesized, we found that high self-reported exposure to the intervention elements significantly increased the correct recall of the social norms messages. Further, our results suggest that girls had a better retention of *The GOOD Life* content than boys, which could be explained by girls being more attentive in class and more willing to participate in alcohol prevention than boys (1). Our findings reflect that the effectiveness of a social norms intervention relies not only on the level of exposure, but also on the level of the pupils' ability to recall and understand the social norms messages delivered. To support pupils' comprehension of school-based prevention programmes, previous research has recommended to actively engage pupils in the intervention through interactive delivery methods such as gamified elements (6, 16). Similarly, a recent study found that a gamified social norms-based intervention enhanced the reduction in alcohol use compared to a standard social norms intervention (32). *The GOOD Life* intervention incorporated different communication elements and strived to actively engage pupils through the “face-to-face” feedback session as well as through the web-application. The study did not provide data on the actual dose of intervention delivery but, some preliminary data indicated that information about web application was distributed to pupils at only five intervention schools, resulting in actual exposure to the web application at only 30% of the intervention schools. However, we observed that boys accessed the web application more than girls (33) and therefore we conclude that including a web application element in SNA interventions can facilitate a higher exposure and retention for boys. Overall, there is a need for further studies to investigate if gamified elements in school-based preventive programmes could increase pupils' interest for the intervention and in turn foster the retention of the core messages to ensure high intervention effectiveness.

Some limitations of this study should be noted. Data were obtained from self-administered questionnaires and therefore over- or underreporting cannot be ruled out. However, the online survey allowed pupils to answer questions on sensitive issues in an anonymous manner and data collected on substance use among university students have shown to be of high quality, when collected via a confidential online survey (34). In addition, recall bias needs to be considered, as the current analyses of self-reported exposure to the intervention could not distinguish between a lack of exposure to the intervention due to lack of offer or due to a lack of interest from pupils in using or paying attention to individual intervention elements (posters and web-application). However, recall bias is not very likely to interfere with our results for overestimation of peer drinking as there is no reason to assume that pupils categorized as overestimating peer drinking are more or less likely to recall the intervention.

The attrition analyses showed that the loss to follow-up was higher among those who drank alcohol at baseline than for those who were abstinent indicating that the analyses for intervention effects provide more conservative effect estimates due to a restricted range of the primary outcome variables among responders. This attrition effect might at least partly explain the lack of an intervention effect on binge drinking as primary outcome. Also, with a response rate of 58% selection bias cannot be ruled out. However, the effect of individual selection bias may be limited because the attrition mainly occurred at school and class level. Further, only one follow-up survey 3 months after baseline restricts the analysis to short-term effects, which may not persist over time. Due to the short follow-up period, we also missed the opportunity to draw conclusions on any delay in onset of alcohol use among non- and light-drinkers or any longer term effects on norm perceptions or binge drinking.

## Conclusion

Despite these limitations, the present study provides new insights into how exposure to the intervention and delivery changes the effect of a school-based social norm intervention for adolescents. Implementers should assure a high level of exposure to normative feedback messages through different communication channels or intervention elements. Also, we conclude that a good recall of messages is important for the effectiveness of the programme, which in turn is improved, if pupils like the intervention.

## Data Availability

The datasets collected and/or analyzed during the current study are available from the corresponding authors upon reasonable request.

## Ethics Statement

This study was carried out in accordance with the recommendations of the Ethical Committee of the Region of Southern Denmark. The trial was approved by the Ethical Committee of the Region of Southern Denmark (Project-ID: S-20140185). Active parental consents were obtained, because the study participants were minors.

## Author Contributions

LV-H was responsible for the data collection and analysis and wrote the first draft of the manuscript. BR contributed by designing the questionnaire. CS is the principle investigator of the study *The GOOD Life* and the lead in its conception and coordination. LV-H and CS developed the intervention and designed the analyses. All authors contributed to the manuscript, critically reviewed its content, and approved the final version before submission.

### Conflict of Interest Statement

The authors declare that the research was conducted in the absence of any commercial or financial relationships that could be construed as a potential conflict of interest.

## Abbreviations

ELM: Elaboration Likelihood Model
ESPAD: European school survey project on alcohol and other drugs
SRS: Student Response System
SNA: social norms approach.
